# An emerging paradigm for the origin and evolution of shelled amoebae, integrating advances from molecular phylogenetics, morphology and paleontology

**DOI:** 10.1590/0074-02760200620

**Published:** 2021-08-13

**Authors:** Daniel JG Lahr

**Affiliations:** 1 Universidade de São Paulo, Instituto de Biociências, Departamento de Zoologia, São Paulo, SP, Brasil

**Keywords:** microbial evolution, microbial ecology, microbial palaeontology, microbial fossils, ancestral state reconstruction

## Abstract

The phylogenetic paradigm of eukaryotic evolution has changed dramatically over the past two decades, with profound reflections on the understanding of life on earth. Arcellinida testate (shelled) amoebae lineages represent some of the oldest fossils of eukaryotes, and the elucidation of their phylogenetic relationships opened a window to the distant past, with important implications for understanding the evolution of life on earth. This four-part essay summarises advances made in the past 20 years regarding: (i) the phylogenetic relationships among amoebae with shells evolving in concert with the advances made in the phylogeny of eukaryotes; (ii) paleobiological studies unraveling the biological affinities of Neoproterozoic vase-shaped microfossils (VSMs); (iii) the interwoven interpretation of these different sets of data concluding that the Neoproterozoic contains a surprising diversity of organisms, in turn demanding a reinterpretation of the most profound events we know in the history of eukaryotes, and; (iv) a synthesis of the current knowledge about the evolution of Arcellinida, together with the possibilities and pitfalls of their interpretation.

The evolutionary affinities of shelled amoebae in contemporary eukaryotic systematics

The largest group of amoebae with shells, the Arcellinida, are single-celled, sexual eukaryotic microorganisms that originated at least 730 million years ago (Fig. 1). The previous sentence would be considered completely incorrect when the last comprehensive monograph on this group of amoebae was produced, two decades ago.^1^ Since then, what has changed is the evolutionary paradigm for eukaryotic microorganisms (popularly referred to as “protists”). In 2002, Dr Ralf Meisterfeld published the monograph entitled “Order Arcellinida”, on the occasion of the publication of the book “Illustrated Guide to the Protozoa”. This work was promoted by the Taxonomy Commission formed by the International Society of Protistologists (ISoP, at the time still named Society of Protozoologists), being widely considered as one of the most authoritative and comprehensive consensus on eukaryotic microorganisms.

**Fig. 1:**
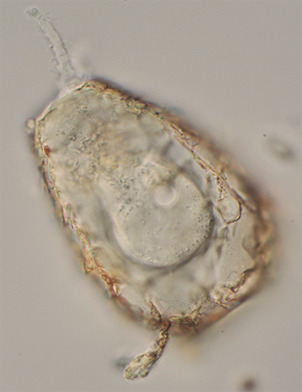
testate amoeba of the genus *Schoenbornia*. The cell of a testate amoeba is completely covered by a shell, in this case composed of agglutinated mineral grains, with only one opening (at the top of the Figure, facing left), through which the pseudopods come out. Pseudopods are the transparent, lobed projections of cytoplasm used by amoebae to both move and feed. Image: DJG Lahr.

The stimulus for launching this great compendium came from the scientific context of the time, when the biological community increasingly understood the evolutionary and ecological importance of eukaryotic microorganisms. Until 2002, amoebae, as well as most eukaryotic microorganisms had been studied in detail only under the morphological aspect. Both naked amoebae or those that bear a shell ― shells are called “tests” in specialised literature, as such the organisms are also called testate amoebae or shelled amoebae, I consider these terms synonymous ― were treated accordingly. As microorganisms have few morphological characteristics, complex and deep systematisations of the different groups were not possible simply because of the lack of characters.^2^ However, the evolutionary landscape began to be unveiled during the 1990’s, when genetic sequencing technologies became more popular and began to be applied to eukaryotic microorganisms. These advances have brought great attention from the scientific community to microorganisms in general, and this intense search for information required an evaluation of the state-of-the-art of protistological science. The hypothesis of the three domains (Bacteria, Archaea and Eukarya) has been around the literature since the 1970’s, but it was only in the early 1990’s that the notion was widely accepted.^3^

Concomitantly, the first comprehensive phylogenies of eukaryotes emerged, and demonstrated that the relationship among organisms was much more complex than the traditional Five Kingdoms preached.^4^ The molecular studies were initially based on analysis of the sequence of the small subunit of the ribosome (18s, or SSU-rDNA), and brought limited resolution to the different eukaryotic lineages. With the addition of universal and conserved genes, such as those that encode the cytoskeletal proteins actins and tubulins, the main relationships among eukaryotes became clearer. The fundamental work in sedimenting this knowledge determined, among other things, that fungi and animals were more closely related to each other than any of them to plants.^5^ This fact came with great surprise from the general scientific community, but only solidified a view that was already common-place among protistologists.^2^ In subsequent years, accompanying the popularisation of sequencing techniques, into the current techniques of high-throughput sequencing age, more microorganisms were sequenced and more genes incorporated into the historical reconstructions, eventually reaching the current understanding of phylogenetic relationships among eukaryotic microorganisms^6^ (Fig. 2).

**Fig. 2:**
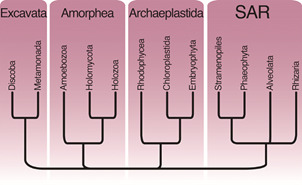
evolutionary relationships among eukaryotic organisms. This figure is based on the most recent proposal for the classification of eukaryotic organisms, carried out by a consortium of about 50 specialists in the different groups. This current classification clarifies for the broad community where the mascroscopic groups are (such as Plants / Embryophyta, Animals / Holozoa and Fungi / Holomycota). Based on work by Adl and collaborators published in 2019.^6^

Eukaryotic microorganisms are currently classified into larger lineages popularly and informally called “Supergroups”: within Amorphea, Amoebozoa gathering most of the organisms capable of producing lobed pseudopods (including Arcellinida testate amoeba) and Opisthokonta gathering Metazoa and Fungi, in addition to other residual lineages; SARs comprising Stramenopila (or Heterokonta, represented mainly by diatoms), Alveolata (represented by dinoflagellates, ciliates and apicomplexans), and Rhizaria (represented by organisms that produce filamentous pseudopods, mainly foraminifera and Cercozoa); the Excavata bringing together a large part of the flagellated heterotrophs, in addition to the Euglenozoa and several parasitic lineages (e.g., Giardia); and finally the Archaeplastida gathering a large part of the photosynthetic organisms including red, green algae and plants. In addition, there are a number of other minor lineages that are difficult to locate on the tree, such as cryptophytes and haptophytes. The understanding of this phylogenetic complexity put the system of eukaryotic microorganisms in direct opposition to the traditional Linnean nomenclatural system: it was clear that the roots of eukaryotic lineages are much deeper and nested than the “Kingdoms, Phyla, Classes” system could support. So the informal “supergroup” convention was created, eventually abandoned, and there are currently attempts to reconcile the two systems.^6^ ^,^ ^7^ ^,^ ^8^

*Shelled amoebae emerged many times in the history of life* - The first studies on shelled amoebae were published in the 1990’s and early 2000’s, focusing on placing the euglyphid testate amoebae in the eukaryotic tree.^9^ ^,^ ^10^ The first comprehensive molecular phylogeny of the largest group of shelled amoebae, Arcellinida, was only published in 2005.^11^ The main objective at this point was to localise this important group of shelled amoebae within the new organisation of eukaryotic microorganisms. Within the conception of the “Five Kingdoms”, all the ameboid organisms were classified in the Protista, within the Rhizopoda. With the new organisation in super groups, several lineages were recognised with representatives capable of producing pseudopods, and the shelled amoebae were then “homeless” taxonomically for some years. The work of Dr Nikolaev and collaborators has demonstrated without a doubt that the Arcellinida amoebae were within the Tubulinea, a group of Amoebozoa that gathers amoebae such as *Amoeba proteus*, the most popular representative that appears illustrated in practically all high school biology textbooks on the planet.

The placement of Arcellinida within the Tubulinea was quite well received, as it was what was predicted by the morphology: the Arcellinida produce lobed pseudopods ultrastructurally identical to that of the Tubulinea. However, Arcellinida are not the only shelled amoebae: the second largest group, the Euglyphida, had already been located in what would eventually be called Rhizaria, in one of the first molecular publications using protists in the 1990s,^9^ but it was only heavily studied in the 2000s^10^ and fully settled almost twenty years later.^12^ The third group of shelled amoebae, already much smaller than the previous two, the Amphitremida, were determined to belong to Stramenopila also in the 2010’s.^13^ Finally, the Coricida were recently removed from within Arcellinida.^14^ In this way, the expectation of polyphyly of the shelled amoebae, already widely discussed by several authors only in the morphological scope, was strongly confirmed with molecular studies (Fig. 3).

**Fig. 3:**
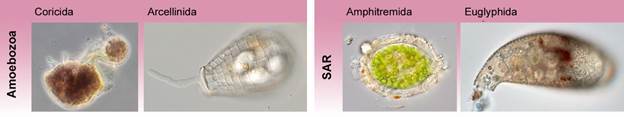
the major groups of shelled amoebae are polyphyletic in the eukaryotic tree. The Euglyphida (with filose pseudopodia) are located in the Stramenopila, Alveolata, and Rhizaria (SAR), as are the Amphitremids. Arcellinida and Coricida are in Tubulinea, in Amoebozoa. The last common ancestor among all shelled amoebae is the last common ancestor of all eukaryotes, so the shelled habit of life emerged independently at least four times. Based on work published by Kosakyan and colleagues in 2016,^8^ images by DJG Lahr.

At the end of the first decade of the 2000’s, with their positions on the tree of life of eukaryotes well established, the relationships among the less inclusive lineages of shelled amoebae and their evolutionary origins remained unresolved. Arcellinida have an estimated diversity between 800 and 2,000 species,^8^ thus being possibly the largest group within Amoebozoa. Historically they had been classified according to the composition of the shell: Difflugiina with agglutinated shells, Arcellina with organic shells and Phryganellina with mixed shells. As studies based on molecular sequences became more comprehensive, including a greater diversity of Arcellinida and a greater number of genes, this arrangement proved to be inaccurate. Further work with increased molecular data and taxonomic coverage indicated that the lineages of shelled amoebae are organised more in accordance with the general shape than with the composition of the shell.^15^ This work already pointed out the need to reevaluate the deep classification of Arcellinida, and several other less inclusive studies corroborated part of the findings (eg., Cavalier-Smith^16^).

Despite the strong evolutionary trend relating systematics to general shell morphology, some other taxonomic issues were still unresolved. With increase in taxonomic sampling, several genera that were quite robust morphologically appeared in reconstructions as non-monophyletic, and importantly, the monophily of the group as a whole began to be contested. Reconstructions using SSU-rDNA and cytoskeletal genes (actin and tubulin) consistently resulted in reconstructions where *Heleopera sphagni* appeared quite distant from other Arcellinida, usually nested within other naked amoebae. At this point, the understanding emerged that the amount of genes used was not sufficient to reconstruct the deepest lines of Arcellinida; therefore, the main issues would need to be revealed using large-scale, multi-gene analysis techniques, such as high-throughput sequencing, within the context of phylogenomics.

Microbial eukaryotes enter the Phylogenomic Era

The first major phylogenomic studies, using comprehensive taxonomic sampling of eukaryotic microorganisms, emerged in the mid-2000s.^17^ ^,^ ^18^ ^,^ ^19^ These studies compiled dozens of taxons and hundreds or thousands of genes and helped solidify the existence of eukaryotic major lineages (named “supergroups” in some circles). More profoundly, they also allowed to unveil the relationships among the supergroups, this aspect was still quite nebulous using phylogenetic reconstructions based on a handful of genes. With the dissemination of knowledge obtained from phylogenomics and the popularisation of methods for obtaining Big Data, the techniques began to be applied to less inclusive groups. Similarly, phylogenetic studies in shelled amoebae were able to demonstrate that the morphological conception of evolutionary relationships, based on shell composition, was not correct. However, the available data did not have sufficient depth to: (1) establish which would be the main deep lineages of shelled amoebae; (2) determine the phylogenetic relationship among them. Due to the successful results in other groups of eukaryotic microorganisms, it was clear that the direction to be taken was to use phylogenomic data; however, several experimental challenges would need to be overcome.

Obtaining phylogenomic data from shelled amoebae presented two major challenges. The first, the lack of annotated genomes closely related to the Arcellinida group. Within Amoebozoa, only *Dictyostelium discoideum* and *Entamoeba histolytica* have complete and well annotated genomes.^20^ ^,^ ^21^ Both organisms are phylogenetically distant from Arcellinida, so the sequencing of genomes would at that time be extremely costly and laborious to answer phylogenetic questions. The alternative, therefore, would be the sequencing of transcriptomes, which can be assembled de novo without a reference genome. However, obtaining transcriptomes of eukaryotic microorganisms leads us to the second great challenge: the vast majority of testate amoebae are unculturable. Therefore, the objective was defined on its own: it would be necessary to obtain transcriptomes from one or a few cells isolated directly from nature.

These types of data were generated firstly for a phylogenomic reconstruction for Amoebozoa in 2017.^14^ In phylogenetic analysis using 61 taxons and 325 genes, the main Amoebozoa lineages were established (Evosea, Tubulinea and Discosea), as well as the phylogenetic relationship among them. The data were obtained from “single-cell transcriptome sequencing”, a technique adapted from a revolutionary method developed for studies with cancer cells.^22^ This technique consists of isolating a single cell directly from nature or a culture, decontaminating it through successive transfers in sterile water, photo-documenting it to create a voucher, lysing the cell, extracting messenger RNA, constructing double-stranded cDNA through reverse transcription, and finally, sequence all the material using high-throuhput techniques.

The success of this technique allowed its application to shelled amoebae, finally unraveling their main deep lineages and the relationships among them.^23^ In agreement with the initial expectations brought by molecular data, the phylogenomic study based on 19 taxa and 250 genes corroborated that the general shape of the shell is the morphological character that most closely reflects diversification of lineages (Fig. 4). This study therefore generated a complete new classification of Arcellinida, dividing them into three major fundamental lineages (Organoconcha, Glutinoconcha and Phryganellina), the most rich in lineages being Glutinoconcha, then subdivided into Sphaerothecina, Longithecina, Excentrostoma, Hyalospheniformes and Volnustoma. Of all the lineages, only Phryganellina, Sphaerothecina and Hyalospheniidae had already been described. The complete restructuring of the Arcellinida’s deep phylogeny demanded a re-evaluation of the understanding of the group’s evolution and diversification.

**Fig. 4:**
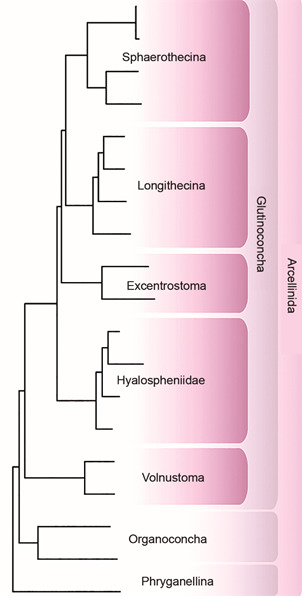
phylogenomic reconstruction of Arcellinida, based on 250 protein coding genes, based on work published by Lahr and collaborators in 2019.^23^

Were there shelled amoebae in the Neoproterozoic oceans?

The Arcellinida have one of the richest, oldest and well-documented fossil records among eukaryotic microorganisms. In the area of micropaleontology, this sentence would also be considered misleading, or at least controversial, about two decades ago - the consensus in 2002 was that the fossil record of shelled amoebae dated, at most, to the Triassic.^1^ Microfossils today understood to be related to Arcellinida are generally referred to as vase-shaped microfossils (VSM). VSMs date mainly from the Neoproterozoic era (1 billion years ago - 541 million years ago) and are concentrated in the Tonian and Cryogenian periods. The Neoproterozoic era predates immediately the Paleozoic, the event of separation between the two being the well-known Cambrian Explosion. Neoproterozoic is divided into three periods: the Tonian (1ba-720ma), the Cryogenian (720ma-635ma) and the Ediacaran (635ma-541ma). The Neoproterozoic is an era marked by important geological events: the breakdown of the super continent Rodinia starting at 900ma, the deep oxygenation of the oceans at about 800ma, two events of extensive glaciations, where possibly the polar ice caps touched the equator, the Sturtian (720ma) and Marinoan (630ma), and finally the great diversification of macroscopic life during Ediacaran. In this way, these fossils are among the oldest fossils undoubtedly eukaryotic, together with acritarcs and chitinozoans *Tappania* and algae-like *Bangiomorpha*.

The argument used by paleobiologists to justify the eukaryotic nature of the fossils revolves around the morphological complexity presented: these microfossils would originate from hard parts produced by cells that had a high degree of cytoskeletal refinement. They would be able to secrete cysts, shells or cell walls with refined ornamentations.^24^ This degree of refinement cannot be achieved with the cytoskeletal machinery of bacteria or archaea. In the case of *Tappania* and other Paleoproterozoic acritarcs, one can only suggest an eukaryotic affinity, however, the structures have very few morphological characteristics to determine affinity for a supergroup, or even more specific eukaryotic lineages. The incredibly detailed *Bangiomorpha*, on the other hand, is clearly a red alga.^25^ However, in the case of *Bangiomorpha*, the problem lies in determining its exact age ― stratigraphy only restricts its age between 1.8ba - 700ma, that is, it may be one of the oldest eukaryotes, or as recent as Neoproterozoic fossils. Tonian VSMs, on the other hand, have rich ornamentation and over the past few decades have been extensively studied.

VSMs have been known to paleobiologists for many decades.^26^ Initially described as acritarcs or chitinozoa by several researchers, they were deeply studied in their morphology, taphonomy and stratigraphy in the 1980’s by Dr Bonnie Bloeser. The American researcher produces the first comprehensive description for VSMs, placing them in a new Genus *Melanocyrillium*.^27^ The fossils were found in shales in the Kwagunt formation (Chuar Group, Grand Canyon), and are exceptionally well preserved specimens, maintaining all the fundamental morphological characteristics: the general shape of the shell, the unique opening and the shell ornaments. Dr Bloeser efficiently argues that these microfossils are significantly distinct from chitinozoans, as they have very distinct, ornate (triangular or hexagonal) openings. At this point, Dr Bloeser interpreted these fossils as possible cysts, and the openings would be the excision mechanism. It was not possible at the moment to make any suggestion of affinity more specific than “eukaryote”.

At the beginning of this millennium, with renewed interest in the evolution of eukaryotic microorganisms, there was also a resurgence in studies with microfossils. The year 2000 is marked by two fundamental studies, which deepened both the morphological understanding and the diversity of VSMs. One study by Drs. Susannah Porter and Andrew Knoll demonstrated that VSMs were much more diverse, prevalent and abundant than previously thought.^28^ Still working on the Kwagunt formation, but studying different types of rocks (including dolomite, dolomicrite and dolosiltite), these researchers described hundreds of VSMs, many falling within the three species of the *Melanocyrillium* genus, originally described by Dr Bloeser, but there were many other morphologies present. Importantly, the researchers suggest: (1) an affinity with shelled amoebae, based on the general morphology (thin, hollow shells, with only one opening, with organic material in its composition); (2) taphonomic explanations for the different preservation modes found, fundamentally different types of mold formation. In the same year, Drs Mónica Martí-Mus and Małgorzata Moczydłowska studied Neoproterozoic VSMs from carbonates of the Visingsö group, in Sweden.^29^ These researchers focused deeply on the morphological aspect, using several advanced imaging techniques (EDX X- ray energy dispersive analysis and confocal microscopy), to obtain more information that would lead to a biological affinity with modern groups, also to obtain interpretations for the possible taphonomy of these fossils. In the taphonomic question, these researchers draw conclusions similar to Porter and Knoll.^28^ However, they do not indicate a conclusive biological affinity, renewing the previous dillema that a relation was possible to algae, foraminifera, tintinid ciliates or shelled amoebae. These two works were fundamentally important to establish that: (1) VSMs are an abundant source of information, with taphonomy now well understood; (2) VSMs have a possible global distribution; and (3) Tonian VSMs have specific characteristics, and must be treated separately from acritarcs or chitinozoa.

With the taphonomic question more deeply understood, there were still doubts about the biological affinity of VSMs. Are these stem groups, completely extinct? Or are they related to groups that still have modern members? The most important work in this regard was published in 2003, by Drs Susannah Porter, Ralf Meisterfeld and Andrew Knoll.^30^ In this work, paleobiologists Porter and Knoll joined with the specialist in shelled amoebae Ralf Meisterfeld, and they compare a series of morphological data on modern shelled amoebae and VSMs. Importantly, shells with only one chamber and one opening exclude the possibility of a foraminiferan affinity ― although there are uniloculated foraminifera, they have other morphological differences and have a well-determined and much more recent fossil record.^31^ The thickness of the shells is inconsistent with a tintinid affinity, and they must also be much more recent. Uni or multicellular algae cysts have a distinct morphology from these shells, and are never agglutinated. Therefore, the most plausible interpretation is that the Tonian VSMs are related to the shelled amoebae. Here it is crucial to remember that “shelled amoebae” is a highly polyphyletic term (Fig. 3). Although still not well established how far apart the shelled amoebae would be in the eukaryotic tree, Dr Meisterfeld understood the possibilities very well and treated the matter carefully. The authors therefore describe in detail the morphology of the different fossils found in greater abundance, and provide a new taxonomic classification. They describe nine new species and eight new genera, including the original *Mellanocyrilium* now divided in three parts, with the new *Cycliocirillium* and *Bonniea*. The morphological argument was therefore crucial in advancing the taxonomic study of the group, and the possible biological affinities were restricted to two modern groups: Arcellinida and Euglyphida, but without more specific affinities. Further details were impossible to be determined, since at the time the understanding of the relationships among shelled amoebae was extremely poor, the entire area of molecular reconstruction of eukaryotes was yet to be developed. This work is perhaps the main milestone in understanding of the evolutionary relationships of amoebae with shells, since it represents the apotheosis of all the knowledge generated so far, and makes it clear that any progress would require advances in both paleontology and systematics.

The advances made in the area of molecular reconstruction have already been discussed in the first section of this essay. From 2003 to 2009, the VSM paleontology area faced a period of inactivity with resurgence in the decade of 2010. In 2011, Dr Tanja Bosak described microfossils congruent with VSMs, but in another geological period and with very different characteristics.^32^ The VSMs were found in carbonate layers of Namibia and Mongolia, whose stratigraphy places them in the Cryogenian period; that is, exactly after the Tonian. This discovery is interesting because in addition to representing one of the few fossil records of life during the Cryogenian, it also increases the importance of the Tonian record because representatives are being found here that continue the record of VSMs, giving strength to the interpretation that they are organisms possibly related to other modern taxa, and not a fossil group with no extant relationships. Cryogenian VSMs underwent a very distinct taphonomic process of preservation from those in Tonian strata. However, several characteristics of the preserved shells have strong indications of affinities with shelled amoebae. Of these, I highlight the presence of particles agglutinated in an originally organic matrix. Within the same shell, one can find particles composed of amorphous silica (consistent with shelled amoebae that biomineralise their own shell) and also aluminum-silicate particles (consistent with shelled amoebae that capture particles in the environment to produce the shell). The presence of both types of particles is consistent with amoebae of the Netzeliidae family, which, as far as I know, are the only ones capable of doing both types of processes simultaneously. This work therefore expands the registration of VSMs into the Cryogenian, and was followed by several other descriptions, with material from many different locations, corroborating interpretations.^33^ ^,^ ^34^ ^,^ ^35^

The Tonian period also received new descriptions from different locations around the globe.^36^ ^,^ ^37^ ^,^ ^38^ ^,^ ^39^ ^,^ ^40^ The additional findings led to the current taxonomic status of the Neoproterozoic VSMs, which have a total of 14 described genera. At least five taxa have been confidently described for 8 locations distributed around the globe. This solidity of the data has even allowed a current discussion about using *Cycliocyrillium* simplex as a fossil index of the Tonian period.^40^ The findings in the fossil record point to a solid biological affinity with the Arcellinida. The Arcellinida in turn received phylogenomic treatment and their main phylogenetic structures were unveiled. In the next section, we will understand how the information from the two fronts were integrated to illuminate the evolution of microbial eukaryotes in the Neoproterozoic.

VSMs as Arcellinida are a fundamental calibration point for eukaryotic molecular clock studies

Studies with molecular clocks have a long and controversial history. The fundamental work in the field, a classic published in 1962 by Émile Zuckerkandl and Linus Pauling,^41^ and subsequently expanded by the same authors in 1965,^42^ was responsible for substantiating the Neutral Theory of Molecular Evolution. Different authors then proposed this theory almost simultaneously: in 1968 by Motoo Kimura,^43^ and independently in 1969 by King and Jukes.^44^ Theory postulates that: the vast majority of mutations occurring in populations have no adaptive value (positive or negative). In this way, they are only subject to processes of genetic drift. Therefore, the effective size of the population almost exclusively determines the fixation or deletion of mutations, in a fully stochastic manner. The proposal was initially understood as strongly opposed to the natural selection mechanism widely accepted by most evolutionary biologists. A long debate between the “selectionists” versus the “neutralists” followed, but eventually both views found a way to live together.^45^

The methods of historical reconstruction based on gene sequences, and the rudimentary methods of implementing molecular clocks were highly controversial in its time; and remained so during subsequent decades,^46^ Zuckerkandl and Pauling’s proposal was that the substitutions found in genes always occurred within a certain period of time, which would allow using the number of substitutions to calculate the genetic distance among species ― that is, mutations occur just like a clock ticks time with its tick- tock. If the temporal distance among species is also known, a substitution/time function could be created, which would allow extrapolating to other cases within the context of a phylogenetic tree. In subsequent years, due to the great interest in this area of study, it was understood that: not all genes actually follow a molecular clock; there may be variation among taxa (even very closely related); the “ticking” rate of the clock can vary over time; among many other mishaps.^47^ With the advancement of computational power, and the concomitant development of more refined algorithms in the area of bioinformatics, the so-called “relaxed molecular clocks” (RMCs) eventually emerged. These implementations of the Zuckerkandl and Pauling idea allow to explicitly parametrise all the biases that can interfere in the calculation of the molecular clock, especially those related to the rate variation over time, and along branches.^48^

With the resurgence in studies of eukaryotic microorganisms, several researchers have looked into the application of molecular clocks to the vast amount of molecular data being generated. There have been attempts to apply the clock techniques to some specific groups, using one or a few genes, such as for ciliates,^49^ and foraminifera,^31^ yet others have used phylogenomic data and extensive sampling.^17^ However, these early efforts found serious divergences with the fossil record and classic interpretations of the evolution of these organisms. The problems related to these first attempts are innumerable, going through phylogenetic uncertainty, inadequate calibrations, inadequate clock models, phylogenetic artifacts, among others (the subject was thoroughly reviewed by Andrew Roger and Laura Hug^50^).

Greater numbers of genes and taxa became available during the first decade of this century. In addition, a series of uncertainties about the study of eukaryotic microorganisms have been resolved, as discussed in the first section of this essay. In this way, studies with molecular clocks became more congruent and illuminating. Two large, widely accepted studies used different methods to obtain similar data: (1) the first used a matrix of about 40 thousand amino acids, but only 36 eukaryotic taxa;^51^ the second used only the SSU-rDNA, but a large taxonomic sample with about 240 eukaryotic taxa.^52^ Both studies have already implemented CMR algorithms and multiple fossil calibrations, to conclude that eukaryotes would have diversified about 1.1 to 1.2 billion years ago. This conclusion was very congruent with the prevailing view in paleontology at the time, which identified the oxygenation of the oceans as the main event that would have driven the emergence of eukaryotes.^24^

However, new geochemical discoveries, and increased taxonomic and genetic sampling of eukaryotes, would push these dates into the more distant past. The first study to be widely accepted was published in 2011 by Laura W Parfrey and collaborators.^53^ Using more than a dozen genes and more than a hundred eukaryotic taxa, this study represented a midway approach between using too many genes or using too many taxa. In addition, fossil calibrations were well supported by the literature, which led to greater acceptance by the paleobiology community; the use of ambiguous fossils had been common criticism in all previous studies. Importantly, this study indicated that eukaryotes arose long before 1.2 billion years ago, and may even reach 1.8 billion years ago. However, the trees obtained clearly show long branches between the origin of eukaryotes, and the diversification of modern lineages about 1.2 billion years ago. Therefore, this study consolidated the knowledge of fossils with eukaryotic affinities that predate the 1.2 billion years limit pointed out by previous molecular studies. Subsequently, studies expanding both taxonomic and molecular sampling by Parfrey and colleagues have only corroborated these conclusions.^54^ ^,^ ^55^

The shelled amoebae, mainly from the Arcellinida, were fundamental calibration points for the molecular dating of eukaryotes. Representing the only group capable of leaving a fossil record within the entire Amoebozoa Supergroup, and the only unambiguous record outside macroscopic groups (plants, fungi and animals), Arcellinida are typically the oldest fossil calibration present in reconstructions using the molecular clock.^53^ ^,^ ^54^ ^,^ ^55^ Its presence in the Neoproterozoic is therefore fundamental to understand the first phase of the evolution of modern eukaryotes; that is, before the Cryogenian period and after the deep oxygenation of the oceans.^24^ However, the diversity of VSMs found in the Tonian and Cryogenian records still needed further clarification of the phylogenetic relationships of Arcellinida.

With the phylogenomic reconstruction of Arcellinida proposed by my collaborators and myself,^23^ we were able to further analyse the morphological relationships between VSMs and modern testates (Fig. 5). Using Bayesian and Maximum Likelihood techniques, we reconstructed the morphology of the hypothetical ancestors resulting from the phylogenomic analysis. The general morphology of five of the hypothetical ancestors is extremely congruent with species described in the fossil record. The data are so robust that the international community immediately accepted them, and as suggested by Susannah Porter and Leigh Anne Reidman, the Tonian VSM species can be classified within the deep lineages described by the phylogenomic analysis.^56^ This interpretation has a subtle but important consequence: the Tonian period is not the time of origin, neither of diversification for Arcellinida. Since VSMs can be reliably classified into modern groups, this means that the groups were already diversified at least 730 million years ago.

**Fig. 5:**
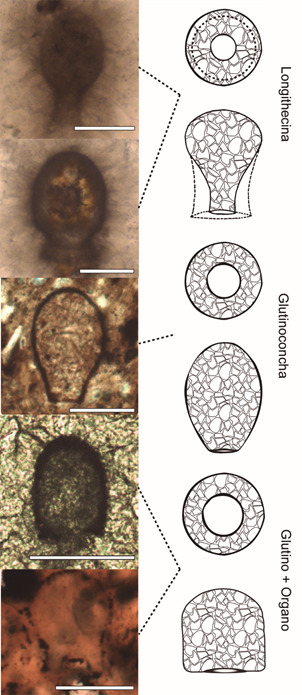
comparison among real fossils (left), and some ancestors reconstructed using phylogenetic methods, on the right. The fossil images are, from top to bottom *Limeta lageniformis*, *Palaeoamphora urucumense*, *Cycliocyrillium torquata*, *Taruma rata* and *Mellanocyrillum hexodiadema*. The scale bars represent 50 µm. The drawings are interpretations of the morphological reconstruction data, and always initially show an apertural view of the hypothetical ancestor, followed by a lateral view. Modified from Lahr et al. in 2019,^23^ images by L Morais.

The moment and environment of origin of the modern Arcellinida

Despite huge advances in the field of eukaryotic evolutionary biology since the last major Arcellinida monograph in 2002,^1^ fundamental questions still need to be answered. All VSMs described for Tonian and Cryogenian, that is, the entire Arcellinida Neoproterozoic fossil record, occur in paleoenvironments interpreted as marine. On the other hand, all modern Arcellinida occur, without exception, in continental environments of freshwater (lakes, rivers, humid soils, mosses, peatlands). I consider, therefore, the main evolutionary link to be solved for Arcellinida is the following:

How are the organisms represented by the Tonian and Cryogenian microfossils related to modern Arcellinida, and when the transition from the marine environment to the freshwater environment occurred.

The issue is subtly complicated. 

First, it is necessary to determine the phylogenetic relationships between microfossils and modern Arcellinida. In this first point, there are fundamentally two possibilities. The first is that VSMs would represent a paraphyletic group from which modern Arcellinida emerged (Panels A and C in Fig. 6, a stem-group relationship). The second is that VSMs would already be part of the “crown-group”, and would relate independently to different modern Arcellinida lineages (Panels B and D in Fig. 6).

**Fig. 6:**
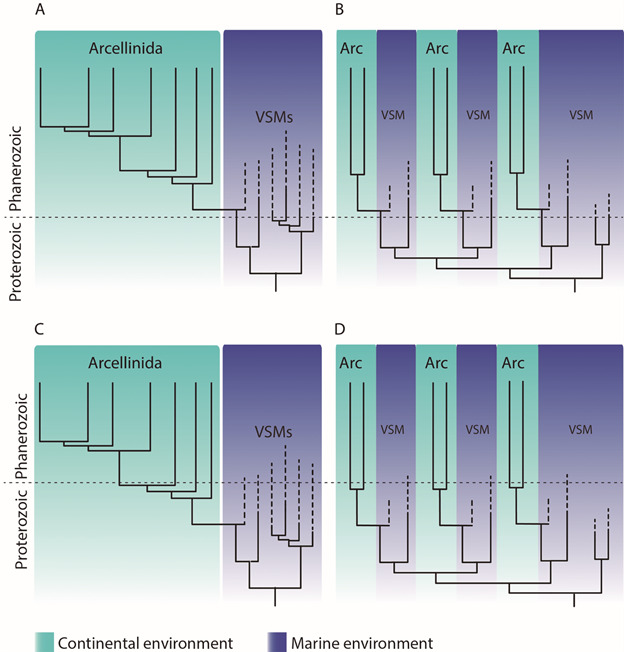
possible interpretations for the evolutionary history of Arcellinida (Arc), with the data currently available. Panel A indicates that vase-shaped microfossils (VSMs) would be a “stem group”, paraphyletic in relation to modern Arcellinida, and the invasion of the continental water environment would have occurred only in the Phanerozoic period. Panel B indicates that VSMs and modern Arcellinida have interrelated phylogenetic relationships, and the invasion of the continental water environment would have occurred only in the Phanerozoic period. Panel C indicates that the VSMs would be a “stem group”, paraphyletic in relation to modern Arcellinida, and the invasion of the continental water environment would have already occurred in the Proterozoic period. Panel D indicates that the VSMs and modern Arcellinida have interrelated phylogenetic relationships, and the invasion of the continental water environment would have already occurred in the Proterozoic period.

This important aspect I call “uncertainty in the interpretation of phylogenetic data”. This uncertainty is based on a very clear, methodological difficulty well-known to those working with the evolution of microorganisms: for fossils, we have only a limited amount of morphological information; for modern organisms, only phylogenomic data is capable of resolving deep relationships. In this way, we can never use the same phylogenetic analysis to test the relationships between fossils and modern organisms. Using the methodology of reconstructing ancestral characters, we can risk a morphological comparison. *In my interpretation*, the results of the reconstruction of ancestral characters support the hypotheses of relationship where VSMs interweave Arcellinida (i.e., panels B and D of Fig. 6). The problem with this methodology is that there are a series of assumptions made in the various stages of the process, from the construction of the phylogenomic tree, through the interpretation and coding of morphological characters, and finally, in the reconstruction of ancestral characters themselves. The general topology provided by phylogenomics is unlikely to be modified, as I have already explained for eukaryotes as a whole, this type of data is extremely robust (Fig. 4). However, with the addition of more taxa ― More than 50% of Arcellinida genera have not yet been molecularly sampled,^8^ ^,^ ^23^ reconstructions of ancestral states can change significantly.

Secondly, the paleoenvironment where we find VSMs determines the moment when invasion of continental waters occurred. There are also two fundamental possibilities here. The first is that VSMs existed only in marine waters, and concomitantly with their extinction, the invasion of continental waters occurred, something that can be interpreted as an “evasion” of the oceans (Panels A and B of Fig. 6). The second is that in the Proterozoic there had already been one or multiple invasions of continental waters, thus coexisting for some time marine and freshwater testates, with all marine lineages subsequently going extinct (Panels C and D of Fig. 6).

This aspect I call the “uncertainty in the interpretation of the fossil record”. VSMs occur in fossiliferous layers that are very difficult to interpret. In addition to intrinsic antiquity, it is still not very well established what exactly were the taphonomic conditions to which the different microfossils were subjected, as discussed above and reviewed by Morais et al.^40^ In the first in-depth study of the VSMs from the Chuar group, there is a discussion as to whether the environment was a shallow area of the sea, or it could be an estuarine region with a strong inhuence of fresh water from river discharge.^28^ However, the current consensus among geologists and paleobiologists is that all fossil layers where VSMs were found are testimonies to marine environments.^56^ Therefore, the registration of VSMs seems to be restricted to the Tonian and Cryogenian periods, strengthening evolutionary scenarios in which the invasion of continental environments would have occurred only in the Phanerozoic, since no VSM was described for Phanerozoic. *This interpretation favored by paleobiologists* supports the evolutionary scenarios demonstrated in A and B (Fig. 6). But an even deeper question regarding the fossil record still exists, an aspect taught to biology students still in high school: the fossil record is incomplete.^57^ Crucially, if any VSM from the marine environment is described for the Phanerozoic, or some VSM from the continental environment is described for the Proterozoic, interpretations C and D are more plausible (Fig. 6).

In conclusion: a paradigm emerges

Combining the interpretations favored by modern researchers both in the area of phylogenetic reconstruction and in the area of paleobiology, the evolutionary scenario that combines all the characteristics is represented in panel B (Fig. 6). In response to the question raised in this section, I end this essay with a synthetic analysis, and I affirm that in light of current knowledge, the organisms represented by the Tonian and Cryogenian microfossils belong to the Arcellinida group, and are related to several less inclusive groups within it. The transition from the marine environment to the freshwater environment occurred multiple times, all in the Phanerozoic.
